# (*E*)-4-[(4-Fluoro­benzyl­idene)amino]-3-[1-(4-isobutyl­phen­yl)eth­yl]-1-(morpholino­meth­yl)-1*H*-1,2,4-triazole-5(4*H*)-thione methanol hemisolvate

**DOI:** 10.1107/S1600536810015217

**Published:** 2010-04-30

**Authors:** Jia Hao Goh, Hoong-Kun Fun, A. C. Vinayaka, B. Kalluraya

**Affiliations:** a X-ray Crystallography Unit, School of Physics, Universiti Sains Malaysia, 11800 USM, Penang, Malaysia; b Department of Studies in Chemistry, Mangalore University, Mangalagangotri, Mangalore 574 199, India

## Abstract

In the title compound, C_26_H_32_FN_5_OS·0.5CH_4_O, the methyl group of the methanol solvent mol­ecule is disordered over two sites with equal occupancies and the solvent is further disordered about a crystallographic twofold rotation axis. The organic mol­ecule exists in a *trans* configuration with respect to the acyclic C=N bond. An intra­molecular C—H⋯S hydrogen bond generates an *S*(6) ring motif. The morpholine ring adopts a chair conformation. The essentially planar 1,2,4-triazole ring [maximum deviation = 0.013 (2) Å] forms dihedral angles of 11.21 (10) and 67.53 (11)°, respectively, with the fluoro­phenyl unit and the isobutyl-substituted benzene ring. The crystal structure is stabilized by a weak inter­molecular C—H⋯π inter­action.

## Related literature

For general background to and applications of 1,2,4-triazole derivatives, see: Calhoun *et al.* (1995 ▶); Pandeya *et al.* (1999 ▶, 2000 ▶); Sujith *et al.* (2009 ▶). For graph-set descriptions of hydrogen-bond motifs, see: Bernstein *et al.* (1995 ▶). For closely related structures, see: Goh *et al.* (2010**a* ▶,b* ▶). For the stability of the temperature controller used for the data collection, see: Cosier & Glazer (1986 ▶).
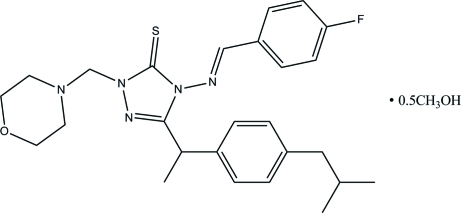


## Experimental

### 

#### Crystal data

C_26_H_32_FN_5_OS·0.5CH_4_O
*M* *_r_* = 497.65
Monoclinic, 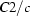

*a* = 40.186 (3) Å
*b* = 4.7840 (3) Å
*c* = 30.073 (2) Å
β = 116.112 (2)°
*V* = 5191.5 (6) Å^3^
*Z* = 8
Mo *K*α radiation
μ = 0.16 mm^−1^
*T* = 100 K
0.31 × 0.16 × 0.06 mm

#### Data collection

Bruker APEXII DUO CCD area-detector diffractometer
Absorption correction: multi-scan (*SADABS*; Bruker, 2009 ▶) *T*_min_ = 0.951, *T*_max_ = 0.990
24705 measured reflections
5921 independent reflections
4378 reflections with *I* > 2σ(*I*)
*R*_int_ = 0.056

#### Refinement

*R*[*F* ^2^ > 2σ(*F* ^2^)] = 0.048
*wR*(*F* ^2^) = 0.149
*S* = 1.04
5921 reflections
337 parameters
H-atom parameters constrained
Δρ_max_ = 0.63 e Å^−3^
Δρ_min_ = −0.22 e Å^−3^

### 

Data collection: *APEX2* (Bruker, 2009 ▶); cell refinement: *SAINT* (Bruker, 2009 ▶); data reduction: *SAINT*; program(s) used to solve structure: *SHELXTL* (Sheldrick, 2008 ▶); program(s) used to refine structure: *SHELXTL*; molecular graphics: *SHELXTL*; software used to prepare material for publication: *SHELXTL* and *PLATON* (Spek, 2009 ▶).

## Supplementary Material

Crystal structure: contains datablocks global, I. DOI: 10.1107/S1600536810015217/lh5026sup1.cif

Structure factors: contains datablocks I. DOI: 10.1107/S1600536810015217/lh5026Isup2.hkl

Additional supplementary materials:  crystallographic information; 3D view; checkCIF report

## Figures and Tables

**Table 1:** Hydrogen-bond geometry (Å, °) *Cg*1 is the centroid of the 1,2,4-triazole ring (N2/C8/N3/N4/C9).

*D*—H⋯*A*	*D*—H	H⋯*A*	*D*⋯*A*	*D*—H⋯*A*
C7—H7*A*⋯S1	0.93	2.51	3.221 (2)	133
C22—H22*A*⋯*Cg*1^i^	0.97	2.64	3.492 (2)	146

Symmetry code: (i) 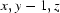.

**Fig. 1.**
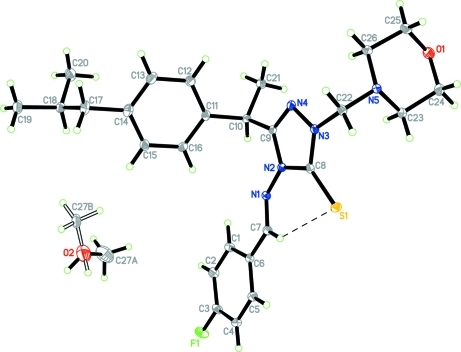
The molecular structure of the title compound, showing 50% probability displacement ellipsoids for non-H atoms and the atom-numbering scheme. An intramolecular hydrogen bond is shown as dashed line. The open bond in the solvent molecule indicates a disordered component.

## supplementary crystallographic information

### Comment

Non-steroidal anti-inflammatory drugs (NSAIDs) are widely used. Despite their large therapeutic applications, they have several undesired, often serious side effects (Calhoun *et al.*, 1995). Therefore, long term administration is not advisable. The need for new anti-inflammatory drugs is obvious and accordingly, there have been renewed interest in anti-inflammatory agents endowed with potent biological activity. In this context, it has been shown that some Mannich bases find applications as anti-inflammatory, analgesic agents (Sujith *et al.*, 2009) and anti-microbial properties (Pandeya *et al.*, 1999, 2000).

The asymmetric unit of the title 1,2,4-triazole compound (Fig. 1) comprises of a (*E*)-4-[(4-fluorobenzylidene)amino]-3-[1-(4-isobutylphenyl)ethyl] -1-(morpholinomethyl)-1*H*-1,2,4-triazole-5(4*H*)-thione molecule and a methanol molecule of crystallization, which is partially occupied with a fixed occupancy of 0.5. The atom C27 of the methanol solvent molecule is disordered over two sites with an equal occupancy of 0.25. Both the disordered components are further disordered over a crystallographic two-fold rotation [symmetry code to generate equivalent atoms: -x, y, -z + 1/2]. The main molecule exists in an *E* configuration with respect to the acyclic C7═N1 double bond [bond length of C7═N1 = 1.276 (2) Å and torsion angle of C6–C7–N1–N2 of 176.41 (16)°]. An intramolecular C7—H7A···S1 hydrogen bond generates a six-membered ring, producing an *S*(6) ring motif (Bernstein *et al.*, 1995). The morpholino unit adopts a chair conformation, with puckering parameters of Q = 0.580 (2) Å, θ = 178.2 (2)° and φ = 126 (6)°. The 1,2,4-triazole ring (N2/C8/N3/N4/C9) is essentially planar, with maximum deviation of -0.013 (2) Å for atom N2. The 1,2,4-triazole ring is inclined at dihedral angles of 11.21 (10) and 67.53 (11)°, respectively, with fluorophenyl group (C1-C6/F1) and isobutyl-substituted benzene ring (C11-C16). The bond lengths and angles are consistent to those observed in closely related structures (Goh *et al.*, 2010*a,b*).

In the crystal structure, no significant intermolecular hydrogen bonds are observed. The crystal structure is stabilized by a weak intermolecular C22—H22A···*Cg*1 interactions (Table 1) involving the 1,2,4-triazole ring.

### Experimental

A mixture of Schiff base (0.01 mol) and formaldehyde (40 %, 2 ml) in ethanol (15 ml) was taken to this solution morpholine (0.01 mol) was added. The reaction mixture was stirred at room temperature for two days. The solid product obtained was collected by filtration, washed with ethanol and dried. Colourless single crystals suitable for X-ray analysis were obtained from a 1:2 mixture of *N,N*-dimethylformamide and methanol by slow evaporation.

### Refinement

All hydrogen atoms were placed in their calculated positions, with C—H = 0.93 – 0.98 Å, and refined using a riding model with *U*_iso_ = 1.2 or 1.5 *U*_eq_(C). A rotating group model was used for the C19, C20 and C21 methyl groups. The methanol solvent molecule is refined with a fixed occupancy of 0.5. The atom C27 of methanol solvent molecule is disordered over two positions with an equal occupancy of 0.25. Both the disordered components are further disordered over a crystallography two-fold rotation. A short intermolecular H15A···H27E interactions [2.04 Å] is also observed.

### Crystal data

**Table:**

C_26_H_32_FN_5_OS·0.5CH_4_O	*F*(000) = 2120
*M**_r_* = 497.65	*D*_x_ = 1.273 Mg m^−^^3^
Monoclinic, *C*2/*c*	Mo *K*α radiation, λ = 0.71073 Å
Hall symbol: -C 2yc	Cell parameters from 4090 reflections
*a* = 40.186 (3) Å	θ = 2.3–29.3°
*b* = 4.7840 (3) Å	µ = 0.16 mm^−^^1^
*c* = 30.073 (2) Å	*T* = 100 K
β = 116.112 (2)°	Plate, colourless
*V* = 5191.5 (6) Å^3^	0.31 × 0.16 × 0.06 mm
*Z* = 8	

### Data collection

**Table:**

Bruker APEXII DUO CCD area-detector diffractometer	5921 independent reflections
Radiation source: fine-focus sealed tube	4378 reflections with *I* > 2σ(*I*)
graphite	*R*_int_ = 0.056
φ and ω scans	θ_max_ = 27.5°, θ_min_ = 1.1°
Absorption correction: multi-scan (*SADABS*; Bruker, 2009)	*h* = −45→52
*T*_min_ = 0.951, *T*_max_ = 0.990	*k* = −6→6
24705 measured reflections	*l* = −39→36

### Refinement

**Table:**

Refinement on *F*^2^	Primary atom site location: structure-invariant direct methods
Least-squares matrix: full	Secondary atom site location: difference Fourier map
*R*[*F*^2^ > 2σ(*F*^2^)] = 0.048	Hydrogen site location: inferred from neighbouring sites
*wR*(*F*^2^) = 0.149	H-atom parameters constrained
*S* = 1.04	*w* = 1/[σ^2^(*F*_o_^2^) + (0.0761*P*)^2^ + 4.918*P*] where *P* = (*F*_o_^2^ + 2*F*_c_^2^)/3
5921 reflections	(Δ/σ)_max_ < 0.001
337 parameters	Δρ_max_ = 0.63 e Å^−^^3^
0 restraints	Δρ_min_ = −0.22 e Å^−^^3^

### Special details

**Table:**

Experimental. The crystal was placed in the cold stream of an Oxford Cryosystems Cobra open-flow nitrogen cryostat (Cosier & Glazer, 1986) operating at 100.0 (1)K.
Geometry. All esds (except the esd in the dihedral angle between two l.s. planes) are estimated using the full covariance matrix. The cell esds are taken into account individually in the estimation of esds in distances, angles and torsion angles; correlations between esds in cell parameters are only used when they are defined by crystal symmetry. An approximate (isotropic) treatment of cell esds is used for estimating esds involving l.s. planes.
Refinement. Refinement of F^2^ against ALL reflections. The weighted R-factor wR and goodness of fit S are based on F^2^, conventional R-factors R are based on F, with F set to zero for negative F^2^. The threshold expression of F^2^ > 2sigma(F^2^) is used only for calculating R-factors(gt) etc. and is not relevant to the choice of reflections for refinement. R-factors based on F^2^ are statistically about twice as large as those based on F, and R- factors based on ALL data will be even larger.

### Fractional atomic coordinates and isotropic or equivalent isotropic displacement parameters (Å^2^)

**Table:**

	*x*	*y*	*z*	*U*_iso_*/*U*_eq_	Occ. (<1)
S1	0.088287 (13)	0.32232 (11)	0.024927 (18)	0.02279 (15)	
F1	−0.03130 (3)	1.5045 (3)	0.10905 (5)	0.0353 (3)	
O1	0.25668 (4)	0.2919 (3)	0.07215 (6)	0.0265 (3)	
N1	0.09867 (4)	0.7033 (3)	0.12673 (6)	0.0176 (3)	
N2	0.12134 (4)	0.4899 (3)	0.12369 (6)	0.0161 (3)	
N3	0.15083 (4)	0.1724 (3)	0.10553 (6)	0.0172 (3)	
N4	0.17235 (4)	0.2366 (4)	0.15492 (6)	0.0188 (4)	
N5	0.19454 (4)	0.0176 (3)	0.07277 (6)	0.0174 (3)	
C1	0.05201 (5)	1.1031 (4)	0.14003 (8)	0.0219 (4)	
H1A	0.0759	1.0874	0.1654	0.026*	
C2	0.02764 (6)	1.2953 (5)	0.14380 (8)	0.0252 (4)	
H2A	0.0347	1.4080	0.1717	0.030*	
C3	−0.00756 (6)	1.3156 (4)	0.10502 (8)	0.0247 (4)	
C4	−0.01956 (6)	1.1530 (4)	0.06315 (8)	0.0242 (4)	
H4A	−0.0433	1.1730	0.0376	0.029*	
C5	0.00502 (5)	0.9582 (5)	0.06043 (8)	0.0228 (4)	
H5A	−0.0026	0.8424	0.0328	0.027*	
C6	0.04086 (5)	0.9323 (4)	0.09816 (7)	0.0187 (4)	
C7	0.06528 (5)	0.7189 (4)	0.09341 (7)	0.0201 (4)	
H7A	0.0566	0.5971	0.0666	0.024*	
C8	0.11940 (5)	0.3259 (4)	0.08433 (7)	0.0172 (4)	
C9	0.15399 (5)	0.4326 (4)	0.16470 (7)	0.0170 (4)	
C10	0.16652 (5)	0.5789 (4)	0.21346 (7)	0.0176 (4)	
H10A	0.1552	0.7650	0.2069	0.021*	
C11	0.15292 (5)	0.4252 (4)	0.24672 (7)	0.0189 (4)	
C12	0.17420 (6)	0.2223 (5)	0.28014 (8)	0.0248 (4)	
H12A	0.1974	0.1772	0.2824	0.030*	
C13	0.16132 (7)	0.0854 (5)	0.31034 (8)	0.0299 (5)	
H13A	0.1762	−0.0478	0.3328	0.036*	
C14	0.12667 (6)	0.1438 (4)	0.30763 (7)	0.0244 (5)	
C15	0.10542 (6)	0.3483 (5)	0.27433 (7)	0.0253 (5)	
H15A	0.0822	0.3929	0.2720	0.030*	
C16	0.11845 (5)	0.4869 (5)	0.24448 (7)	0.0230 (4)	
H16A	0.1038	0.6235	0.2226	0.028*	
C17	0.11276 (7)	−0.0019 (5)	0.34084 (8)	0.0287 (5)	
H17A	0.1268	−0.1728	0.3532	0.034*	
H17B	0.0871	−0.0539	0.3212	0.034*	
C18	0.11550 (6)	0.1720 (5)	0.38489 (7)	0.0233 (4)	
H18A	0.1010	0.3432	0.3721	0.028*	
C19	0.09868 (7)	0.0110 (5)	0.41376 (9)	0.0335 (5)	
H19A	0.0736	−0.0387	0.3920	0.050*	
H19B	0.0990	0.1258	0.4401	0.050*	
H19C	0.1129	−0.1556	0.4273	0.050*	
C20	0.15534 (6)	0.2551 (6)	0.41852 (8)	0.0357 (6)	
H20A	0.1647	0.3705	0.4004	0.054*	
H20B	0.1703	0.0901	0.4299	0.054*	
H20C	0.1561	0.3570	0.4465	0.054*	
C21	0.20863 (5)	0.6187 (5)	0.23668 (8)	0.0245 (5)	
H21A	0.2149	0.7449	0.2167	0.037*	
H21B	0.2204	0.4415	0.2385	0.037*	
H21C	0.2170	0.6945	0.2694	0.037*	
C22	0.16324 (5)	−0.0479 (4)	0.08182 (7)	0.0190 (4)	
H22A	0.1692	−0.2133	0.1026	0.023*	
H22B	0.1426	−0.0954	0.0504	0.023*	
C23	0.18987 (6)	0.2658 (4)	0.04242 (8)	0.0224 (4)	
H23A	0.1892	0.4312	0.0606	0.027*	
H23B	0.1666	0.2543	0.0126	0.027*	
C24	0.22178 (6)	0.2869 (5)	0.02871 (8)	0.0279 (5)	
H24A	0.2211	0.1285	0.0082	0.033*	
H24B	0.2192	0.4559	0.0096	0.033*	
C25	0.26073 (5)	0.0439 (5)	0.10014 (8)	0.0249 (5)	
H25A	0.2846	0.0452	0.1290	0.030*	
H25B	0.2599	−0.1176	0.0802	0.030*	
C26	0.23025 (5)	0.0222 (5)	0.11653 (8)	0.0230 (4)	
H26A	0.2333	−0.1472	0.1357	0.028*	
H26B	0.2314	0.1807	0.1373	0.028*	
O2	0.00036 (14)	0.8833 (15)	0.27352 (18)	0.091 (2)	0.50
H2OA	−0.0207	0.8835	0.2737	0.136*	0.25
H2OB	−0.0184	0.8489	0.2463	0.136*	0.25
C27A	0.0135 (4)	1.187 (3)	0.2744 (6)	0.068 (4)	0.25
H27A	−0.0076	1.3084	0.2593	0.102*	0.25
H27B	0.0281	1.2435	0.3082	0.102*	0.25
H27C	0.0282	1.1993	0.2566	0.102*	0.25
C27B	0.0229 (3)	0.616 (2)	0.2923 (4)	0.039 (2)	0.25
H27D	0.0065	0.4596	0.2860	0.059*	0.25
H27E	0.0383	0.5891	0.2757	0.059*	0.25
H27F	0.0382	0.6333	0.3272	0.059*	0.25

### Atomic displacement parameters (Å^2^)

**Table:**

	*U*^11^	*U*^22^	*U*^33^	*U*^12^	*U*^13^	*U*^23^
S1	0.0194 (3)	0.0310 (3)	0.0140 (2)	0.0030 (2)	0.00371 (19)	−0.00443 (19)
F1	0.0279 (7)	0.0307 (7)	0.0483 (8)	0.0099 (5)	0.0178 (6)	−0.0043 (6)
O1	0.0207 (7)	0.0301 (8)	0.0299 (8)	−0.0040 (6)	0.0123 (6)	0.0000 (6)
N1	0.0165 (8)	0.0198 (8)	0.0174 (8)	0.0025 (6)	0.0083 (6)	−0.0002 (6)
N2	0.0150 (7)	0.0198 (8)	0.0132 (7)	0.0009 (6)	0.0059 (6)	−0.0014 (6)
N3	0.0156 (8)	0.0230 (8)	0.0142 (8)	0.0007 (6)	0.0075 (6)	−0.0014 (6)
N4	0.0177 (8)	0.0250 (9)	0.0142 (8)	−0.0004 (6)	0.0075 (6)	0.0005 (6)
N5	0.0161 (8)	0.0208 (8)	0.0159 (8)	0.0013 (6)	0.0075 (6)	0.0009 (6)
C1	0.0187 (9)	0.0219 (10)	0.0233 (10)	−0.0005 (8)	0.0075 (8)	−0.0008 (8)
C2	0.0254 (11)	0.0245 (10)	0.0260 (11)	−0.0019 (8)	0.0115 (9)	−0.0062 (8)
C3	0.0215 (10)	0.0218 (10)	0.0345 (12)	0.0057 (8)	0.0157 (9)	0.0018 (9)
C4	0.0179 (9)	0.0293 (11)	0.0231 (10)	0.0031 (8)	0.0068 (8)	0.0049 (8)
C5	0.0204 (10)	0.0277 (11)	0.0192 (10)	0.0008 (8)	0.0078 (8)	−0.0004 (8)
C6	0.0172 (9)	0.0206 (9)	0.0187 (9)	0.0018 (7)	0.0085 (8)	0.0025 (7)
C7	0.0177 (9)	0.0246 (10)	0.0176 (9)	−0.0003 (8)	0.0075 (8)	−0.0021 (8)
C8	0.0169 (9)	0.0190 (9)	0.0177 (9)	−0.0012 (7)	0.0095 (7)	−0.0011 (7)
C9	0.0149 (9)	0.0220 (9)	0.0145 (9)	−0.0005 (7)	0.0070 (7)	0.0028 (7)
C10	0.0168 (9)	0.0208 (9)	0.0139 (9)	0.0007 (7)	0.0056 (7)	−0.0001 (7)
C11	0.0218 (10)	0.0217 (9)	0.0129 (9)	−0.0031 (8)	0.0074 (7)	−0.0045 (7)
C12	0.0277 (11)	0.0263 (11)	0.0235 (10)	0.0067 (8)	0.0140 (9)	0.0010 (8)
C13	0.0408 (13)	0.0260 (11)	0.0250 (11)	0.0077 (10)	0.0165 (10)	0.0055 (9)
C14	0.0328 (11)	0.0226 (10)	0.0187 (10)	−0.0064 (8)	0.0121 (9)	−0.0048 (8)
C15	0.0216 (10)	0.0357 (12)	0.0186 (10)	−0.0042 (9)	0.0089 (8)	−0.0011 (9)
C16	0.0189 (9)	0.0312 (11)	0.0154 (9)	−0.0011 (8)	0.0044 (8)	0.0009 (8)
C17	0.0397 (13)	0.0253 (11)	0.0251 (11)	−0.0080 (9)	0.0179 (10)	−0.0020 (9)
C18	0.0251 (10)	0.0274 (11)	0.0192 (10)	−0.0006 (8)	0.0113 (8)	0.0010 (8)
C19	0.0372 (13)	0.0399 (13)	0.0294 (12)	−0.0025 (10)	0.0200 (10)	0.0032 (10)
C20	0.0314 (12)	0.0520 (16)	0.0233 (11)	−0.0063 (11)	0.0117 (10)	−0.0046 (10)
C21	0.0172 (10)	0.0330 (12)	0.0207 (10)	−0.0027 (8)	0.0059 (8)	−0.0017 (8)
C22	0.0194 (9)	0.0194 (9)	0.0195 (9)	−0.0007 (7)	0.0096 (8)	−0.0033 (7)
C23	0.0211 (10)	0.0268 (11)	0.0211 (10)	0.0020 (8)	0.0108 (8)	0.0040 (8)
C24	0.0252 (11)	0.0366 (12)	0.0249 (11)	−0.0015 (9)	0.0139 (9)	0.0040 (9)
C25	0.0179 (10)	0.0286 (11)	0.0272 (11)	0.0010 (8)	0.0092 (8)	−0.0008 (9)
C26	0.0172 (9)	0.0282 (11)	0.0222 (10)	0.0011 (8)	0.0073 (8)	0.0025 (8)
O2	0.056 (3)	0.165 (6)	0.056 (3)	−0.004 (4)	0.031 (3)	0.012 (3)
C27A	0.056 (8)	0.060 (8)	0.092 (11)	−0.010 (6)	0.037 (7)	−0.019 (7)
C27B	0.038 (5)	0.057 (7)	0.030 (5)	−0.009 (5)	0.021 (4)	0.003 (5)

### Geometric parameters (Å, °)

**Table:**

S1—C8	1.6699 (19)	C16—H16A	0.9300
F1—C3	1.358 (2)	C17—C18	1.527 (3)
O1—C25	1.422 (3)	C17—H17A	0.9700
O1—C24	1.436 (3)	C17—H17B	0.9700
N1—C7	1.276 (2)	C18—C20	1.523 (3)
N1—N2	1.398 (2)	C18—C19	1.523 (3)
N2—C9	1.376 (2)	C18—H18A	0.9800
N2—C8	1.393 (2)	C19—H19A	0.9600
N3—C8	1.354 (2)	C19—H19B	0.9600
N3—N4	1.385 (2)	C19—H19C	0.9600
N3—C22	1.477 (2)	C20—H20A	0.9600
N4—C9	1.304 (3)	C20—H20B	0.9600
N5—C22	1.434 (2)	C20—H20C	0.9600
N5—C23	1.458 (3)	C21—H21A	0.9600
N5—C26	1.459 (2)	C21—H21B	0.9600
C1—C2	1.384 (3)	C21—H21C	0.9600
C1—C6	1.399 (3)	C22—H22A	0.9700
C1—H1A	0.9300	C22—H22B	0.9700
C2—C3	1.385 (3)	C23—C24	1.513 (3)
C2—H2A	0.9300	C23—H23A	0.9700
C3—C4	1.374 (3)	C23—H23B	0.9700
C4—C5	1.387 (3)	C24—H24A	0.9700
C4—H4A	0.9300	C24—H24B	0.9700
C5—C6	1.392 (3)	C25—C26	1.512 (3)
C5—H5A	0.9300	C25—H25A	0.9700
C6—C7	1.466 (3)	C25—H25B	0.9700
C7—H7A	0.9300	C26—H26A	0.9700
C9—C10	1.498 (3)	C26—H26B	0.9700
C10—C11	1.524 (3)	O2—O2^i^	1.402 (10)
C10—C21	1.532 (3)	O2—C27B	1.522 (13)
C10—H10A	0.9800	O2—C27A	1.543 (15)
C11—C12	1.388 (3)	O2—H2OA	0.8503
C11—C16	1.389 (3)	O2—H2OB	0.8499
C12—C13	1.391 (3)	C27A—C27A^i^	1.39 (3)
C12—H12A	0.9300	C27A—H27A	0.9600
C13—C14	1.387 (3)	C27A—H27B	0.9600
C13—H13A	0.9300	C27A—H27C	0.9602
C14—C15	1.392 (3)	C27B—H27D	0.9600
C14—C17	1.513 (3)	C27B—H27E	0.9602
C15—C16	1.390 (3)	C27B—H27F	0.9601
C15—H15A	0.9300		
			
C25—O1—C24	109.65 (16)	C18—C19—H19A	109.5
C7—N1—N2	118.83 (16)	C18—C19—H19B	109.5
C9—N2—C8	108.91 (15)	H19A—C19—H19B	109.5
C9—N2—N1	118.60 (15)	C18—C19—H19C	109.5
C8—N2—N1	132.25 (15)	H19A—C19—H19C	109.5
C8—N3—N4	113.44 (15)	H19B—C19—H19C	109.5
C8—N3—C22	127.08 (16)	C18—C20—H20A	109.5
N4—N3—C22	119.48 (15)	C18—C20—H20B	109.5
C9—N4—N3	104.66 (15)	H20A—C20—H20B	109.5
C22—N5—C23	114.50 (15)	C18—C20—H20C	109.5
C22—N5—C26	115.40 (16)	H20A—C20—H20C	109.5
C23—N5—C26	110.90 (16)	H20B—C20—H20C	109.5
C2—C1—C6	120.26 (18)	C10—C21—H21A	109.5
C2—C1—H1A	119.9	C10—C21—H21B	109.5
C6—C1—H1A	119.9	H21A—C21—H21B	109.5
C1—C2—C3	118.35 (19)	C10—C21—H21C	109.5
C1—C2—H2A	120.8	H21A—C21—H21C	109.5
C3—C2—H2A	120.8	H21B—C21—H21C	109.5
F1—C3—C4	118.77 (19)	N5—C22—N3	116.51 (16)
F1—C3—C2	117.96 (19)	N5—C22—H22A	108.2
C4—C3—C2	123.26 (19)	N3—C22—H22A	108.2
C3—C4—C5	117.56 (19)	N5—C22—H22B	108.2
C3—C4—H4A	121.2	N3—C22—H22B	108.2
C5—C4—H4A	121.2	H22A—C22—H22B	107.3
C4—C5—C6	121.33 (19)	N5—C23—C24	109.50 (17)
C4—C5—H5A	119.3	N5—C23—H23A	109.8
C6—C5—H5A	119.3	C24—C23—H23A	109.8
C5—C6—C1	119.22 (18)	N5—C23—H23B	109.8
C5—C6—C7	118.64 (18)	C24—C23—H23B	109.8
C1—C6—C7	122.11 (17)	H23A—C23—H23B	108.2
N1—C7—C6	118.84 (18)	O1—C24—C23	111.04 (17)
N1—C7—H7A	120.6	O1—C24—H24A	109.4
C6—C7—H7A	120.6	C23—C24—H24A	109.4
N3—C8—N2	102.32 (15)	O1—C24—H24B	109.4
N3—C8—S1	127.05 (15)	C23—C24—H24B	109.4
N2—C8—S1	130.55 (14)	H24A—C24—H24B	108.0
N4—C9—N2	110.62 (16)	O1—C25—C26	110.62 (17)
N4—C9—C10	125.22 (17)	O1—C25—H25A	109.5
N2—C9—C10	124.16 (17)	C26—C25—H25A	109.5
C9—C10—C11	110.73 (16)	O1—C25—H25B	109.5
C9—C10—C21	109.42 (16)	C26—C25—H25B	109.5
C11—C10—C21	113.83 (16)	H25A—C25—H25B	108.1
C9—C10—H10A	107.5	N5—C26—C25	108.87 (17)
C11—C10—H10A	107.5	N5—C26—H26A	109.9
C21—C10—H10A	107.5	C25—C26—H26A	109.9
C12—C11—C16	117.86 (19)	N5—C26—H26B	109.9
C12—C11—C10	121.88 (18)	C25—C26—H26B	109.9
C16—C11—C10	120.27 (18)	H26A—C26—H26B	108.3
C11—C12—C13	120.9 (2)	O2^i^—O2—C27B	96.8 (5)
C11—C12—H12A	119.5	O2^i^—O2—C27A	82.7 (6)
C13—C12—H12A	119.5	C27B—O2—C27A	129.9 (7)
C14—C13—C12	121.3 (2)	O2^i^—O2—H2OA	115.4
C14—C13—H13A	119.4	C27B—O2—H2OA	115.5
C12—C13—H13A	119.4	C27A—O2—H2OA	109.4
C13—C14—C15	117.8 (2)	O2^i^—O2—H2OB	54.0
C13—C14—C17	121.4 (2)	C27B—O2—H2OB	108.9
C15—C14—C17	120.7 (2)	C27A—O2—H2OB	110.6
C16—C15—C14	120.9 (2)	H2OA—O2—H2OB	62.8
C16—C15—H15A	119.5	C27A^i^—C27A—O2	83.1 (7)
C14—C15—H15A	119.5	C27A^i^—C27A—H27A	52.0
C11—C16—C15	121.21 (19)	O2—C27A—H27A	109.7
C11—C16—H16A	119.4	C27A^i^—C27A—H27B	161.2
C15—C16—H16A	119.4	O2—C27A—H27B	109.1
C14—C17—C18	114.54 (18)	H27A—C27A—H27B	109.5
C14—C17—H17A	108.6	C27A^i^—C27A—H27C	78.2
C18—C17—H17A	108.6	O2—C27A—H27C	109.6
C14—C17—H17B	108.6	H27A—C27A—H27C	109.5
C18—C17—H17B	108.6	H27B—C27A—H27C	109.5
H17A—C17—H17B	107.6	O2—C27B—H27D	109.8
C20—C18—C19	110.73 (18)	O2—C27B—H27E	109.7
C20—C18—C17	111.82 (18)	H27D—C27B—H27E	109.5
C19—C18—C17	109.78 (18)	O2—C27B—H27F	108.9
C20—C18—H18A	108.1	H27D—C27B—H27F	109.5
C19—C18—H18A	108.1	H27E—C27B—H27F	109.4
C17—C18—H18A	108.1		
			
C7—N1—N2—C9	−165.16 (18)	N2—C9—C10—C21	−143.00 (19)
C7—N1—N2—C8	21.2 (3)	C9—C10—C11—C12	92.1 (2)
C8—N3—N4—C9	0.2 (2)	C21—C10—C11—C12	−31.6 (3)
C22—N3—N4—C9	−179.21 (16)	C9—C10—C11—C16	−88.2 (2)
C6—C1—C2—C3	−0.8 (3)	C21—C10—C11—C16	148.00 (19)
C1—C2—C3—F1	179.92 (19)	C16—C11—C12—C13	0.1 (3)
C1—C2—C3—C4	0.5 (3)	C10—C11—C12—C13	179.68 (19)
F1—C3—C4—C5	−178.78 (19)	C11—C12—C13—C14	0.9 (3)
C2—C3—C4—C5	0.7 (3)	C12—C13—C14—C15	−1.3 (3)
C3—C4—C5—C6	−1.5 (3)	C12—C13—C14—C17	−179.2 (2)
C4—C5—C6—C1	1.2 (3)	C13—C14—C15—C16	0.6 (3)
C4—C5—C6—C7	178.98 (19)	C17—C14—C15—C16	178.64 (19)
C2—C1—C6—C5	0.0 (3)	C12—C11—C16—C15	−0.7 (3)
C2—C1—C6—C7	−177.7 (2)	C10—C11—C16—C15	179.70 (18)
N2—N1—C7—C6	176.41 (16)	C14—C15—C16—C11	0.3 (3)
C5—C6—C7—N1	176.71 (19)	C13—C14—C17—C18	101.5 (2)
C1—C6—C7—N1	−5.5 (3)	C15—C14—C17—C18	−76.4 (3)
N4—N3—C8—N2	−1.5 (2)	C14—C17—C18—C20	−60.0 (3)
C22—N3—C8—N2	177.79 (17)	C14—C17—C18—C19	176.70 (19)
N4—N3—C8—S1	175.59 (14)	C23—N5—C22—N3	−57.1 (2)
C22—N3—C8—S1	−5.1 (3)	C26—N5—C22—N3	73.4 (2)
C9—N2—C8—N3	2.3 (2)	C8—N3—C22—N5	109.5 (2)
N1—N2—C8—N3	176.42 (18)	N4—N3—C22—N5	−71.3 (2)
C9—N2—C8—S1	−174.70 (16)	C22—N5—C23—C24	−171.03 (17)
N1—N2—C8—S1	−0.6 (3)	C26—N5—C23—C24	56.2 (2)
N3—N4—C9—N2	1.3 (2)	C25—O1—C24—C23	59.4 (2)
N3—N4—C9—C10	−178.07 (17)	N5—C23—C24—O1	−56.9 (2)
C8—N2—C9—N4	−2.4 (2)	C24—O1—C25—C26	−60.8 (2)
N1—N2—C9—N4	−177.45 (16)	C22—N5—C26—C25	170.28 (17)
C8—N2—C9—C10	177.03 (17)	C23—N5—C26—C25	−57.4 (2)
N1—N2—C9—C10	2.0 (3)	O1—C25—C26—N5	59.8 (2)
N4—C9—C10—C11	−89.9 (2)	O2^i^—O2—C27A—C27A^i^	41.1 (11)
N2—C9—C10—C11	90.7 (2)	C27B—O2—C27A—C27A^i^	133.9 (10)
N4—C9—C10—C21	36.3 (3)		

Symmetry codes: (i) −*x*, *y*, −*z*+1/2.

### Hydrogen-bond geometry (Å, °)

**Table:**

Cg1 is the centroid of the 1,2,4-triazole ring (N2/C8/N3/N4/C9).

**Table:**

*D*—H···*A*	*D*—H	H···*A*	*D*···*A*	*D*—H···*A*
C7—H7A···S1	0.93	2.51	3.221 (2)	133
C22—H22A···Cg1^ii^	0.97	2.64	3.492 (2)	146

Symmetry codes: (ii) *x*, *y*−1, *z*.
